# Mr. Sydney Jones

**Published:** 1913-12-20

**Authors:** 


### Obituary.

Mr. Sydney Jones, F.R.C.S., has died at the age of eighty-two. Having studied at St. Thomas's Hospital he was appointed assistant surgeon there in 1860, and became a lecturer on surgical anatomy and ophthalmic surgery. In 1871 he began his twenty years of work as full surgeon. A member of the Council of the Royal College of Surgeons, he was examiner to the Royal College of Physicians and to the University of Glasgow. He edited a pathological catalogue of St. Thomas's Hospital Museum, and was consulting surgeon to three cottage hospitals and to the London Throat Hospital.

